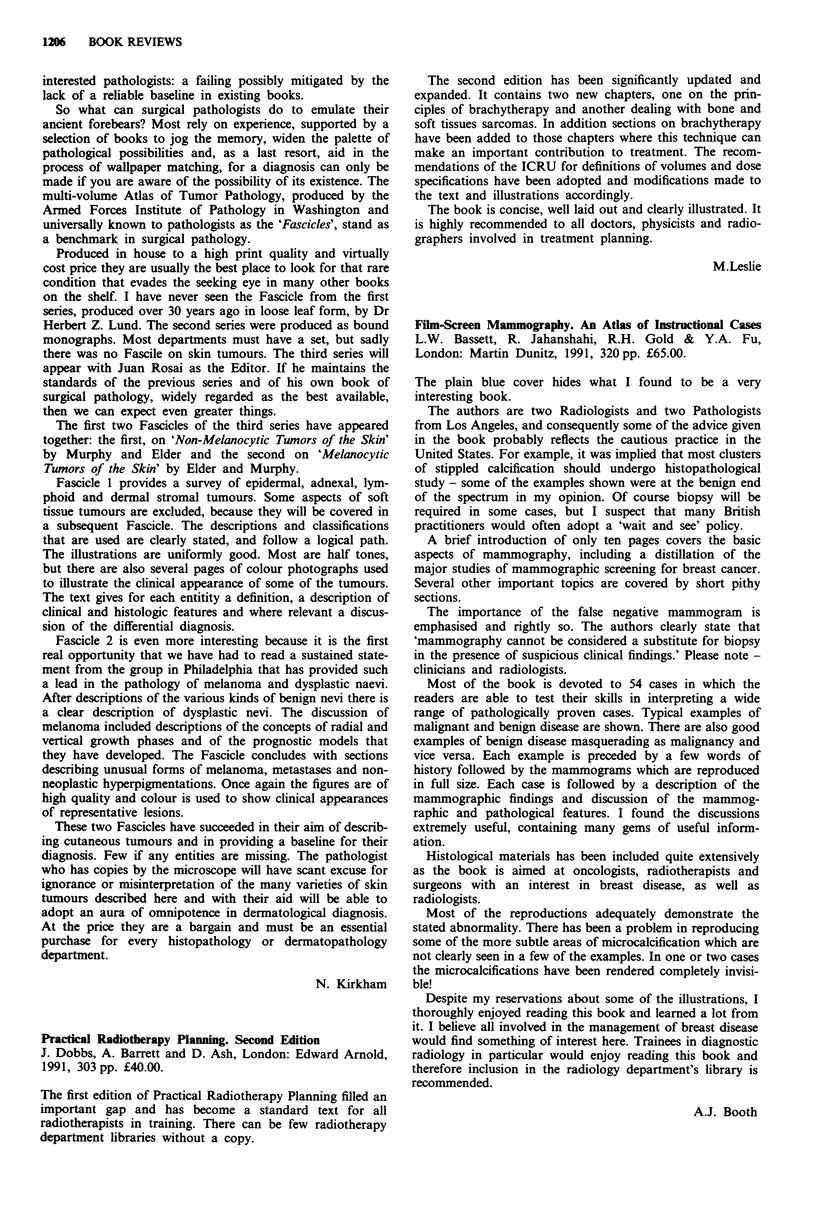# Practical Radiotheraphy Planning (2 ed.)

**Published:** 1992-12

**Authors:** M. Leslie


**Practical Radiotherapy Planning. Second Edition**
J. Dobbs, A. Barrett and D. Ash, London: Edward Arnold, 1991, 303 pp. £40.00.

The first edition of Practical Radiotherapy Planning filled an important gap and has become a standard text for all radiotherapists in training. There can be few radiotherapy department libraries without a copy.

The second edition has been significantly updated and expanded. It contains two new chapters, one on the principles of brachytherapy and another dealing with bone and soft tissues sarcomas. In addition sections on brachytherapy have been added to those chapters where this technique can make an important contribution to treatment. The recommendations of the ICRU for definitions of volumes and dose specifications have been adopted and modifications made to the text and illustrations accordingly.

The book is concise, well laid out and clearly illustrated. It is highly recommended to all doctors, physicists and radiographers involved in treatment planning.

M.Leslie